# Keystone Veterinary Medical Association

**Published:** 1891-05

**Authors:** 


*Keystone Veterinary Medical Association.*—The regular monthly meeting of the Keystone Veterinary Medical Association, was held at the College of Physicians, Philadelphia, February 7, 1891, President, Dr. Hoskins in the chair. Drs. Zuill, Glass, Huidekoper, Weber, Kooker, Eaves, Lusson, Drake, and Schreiber, answered roll call. Minutes of previous meeting were read and adopted, after rectifying the part stating that Dr. Zuill regarded pneumonia as a trifling disease in the horse. The Board of Trustees reported as follows :

"They report unfavorably on the motion made by Dr. Ridge, of the Clinical Staff of the University, Veterinary Department; that the said Clinical Staff be expelled." The report of the Board was accepted. Dr. Hoskins, Chairman of the Legislative Committee, reported having received the report of Counselors Alexander and Magill, regarding the amendments to the Veterinary Bill. In their opinion the law, at this length of time after its passage, is as strong as any amendment could possibly make it. In fact, they have enough confidence in the bill to offer to prosecute a violation of it, to a successful issue, or no fee. Dr. Hoskins, President, announced that for March meeting, that Dr. Huidekoper would read a paper on Meat and Milk Supply of Philadelphia, which would be followed by scientific specialists, expressing their views regarding the effect of diseased meats and milk on the health of the consumers, also the best means for protecting the interests of the dairyman. The April meeting to be devoted to the question, as to how we can procure a uniform examination of veterinary students for graduation.

Under the head of new business Dr. Zuill made some remarks upon the report of the Trustees, and tendered his resignation as a member of the Keystone Veterinary Medical Association, in which he was followed by Drs. Magill, Williams and Ridge. Dr. Magill sent his resignation as secretary, with bill for stationary, etc., all of which were referred to the Board of Trustees. Dr. Glass asked help of the members present for a veterinarian who had become stranded, an old member of the U. S. V. M. A. Dr. Hoskins made the announcement that the next meeting of the United States Veterinary Medical Association, will be held at Washington, on September 22-23, 1891. There were no papers, and on motion adjourned.

W. S. Kooker, *Secretary*, Pro Tem.

---

*Keystone Veterinary Medical Association.*—The regular meeting of the Keystone Veterinary Medical Association was held at the College of Physicians, Philadelphia, March 7, 1891. Dr. Hoskins in the chair. The following members answered roll-call :

Drs. Miller, Huidekoper, Weber, Kooker, Bridge, Lintz, Lusson, R. Formad, Werntz, Drake and Sellers. Present as visitors : Drs. T. B. Rayner, G. B. Rayner, J. W. Gadsden, Frank Standen, Cheston Morris, M. D., P. A. Hummel, J. R. Hart, R. Gladfelter, P. D. Keyes, M. D., State Board of Health, Dr. A. W. Clement of the staff of the Bureau of Animal Industry, of Baltimore, and George Abbott, Esq., a prominent agriculturalist.

On motion by Dr. Kooker, duly seconded and passed, it was agreed that the reading of minutes, and the regular order of business be dispensed with, and that we proceed to the discussion of the Meat and Milk supply of Philadelphia. A letter of regret from Dr. Benjamin Lee, Secretary of the State Board of Health, stating that his absence from the city would prevent his being present, was read by the Secretary as follows :

"The subject of your discussion is one of very great interest to me personally, as well as of the very first importance in its relations to the health of our people. Intelligent foreigners are amazed, when they find how little pains are taken, by our City and State Government, to protect the food supplies of the people. Whatever may be the advantage of a republican form of government, in this respect it has in this country proved itself a miserable failure. Irresponsible and conscienceless persons are permitted to adulterate and poisen our food and water, almost without let or hinderance. The amount of disease and number of premature, and therefore unnecessary deaths, which take place every year in this commonwealth run far up among the thousands. The branch of this subject which your association proposes especially to deal with is one of special magnitude in its relation to infant mortality, and I cannot too heartely commend the earnest spirit in which you are taking it up." Yours, respectfully,

Benjamin Lee, Secretary State Board of Health.

Dr. R. S. Huidekoper was introduced and read a paper on Meat and its Inspection, illustrating his remarks on the blackboard. The earliest inspection of meat is recorded in the Pentateuch, and the code is there laid down, by which to distinguish between animals that are fit for food and those that are not. The Romans had laws for the inspection of meat, but there were no laws elsewhere until very modern times. The large cities of Europe are very far in advance of our own. All large cities of Europe have a rigid inspection. In Bavaria and Switzerland they have a complete veterinary service for the inspection of meat. In Berlin and Paris a large force of veterinarians are employed in slaughter-houses. In Berlin all live stock shipped into the city is unloaded at a certain quarter, where it is examined by veterinarians who have no other duties. Animals are then removed to stables and reinspected and watched for a certain length of time, and are again inspected at the time of slaughter. In the case of hogs portions of each animal are sent to an office where they are examined for trichina and measles. The veterinarians employed as inspectors in the cities of Europe examine all animals in the cattle market, the live animals in the slaughter-houses and abattoirs, examine them after slaughter, and then examine the meat brought into the city that has been slaughtered elsewhere, inspect butcher shops, and inspect all meats furnished prisons and other public institutions.

In this country little or nothing has been done toward inspecting our meat supply. The alterations occurring in meat, which condemn it for use as human food, are those which are due to emaciation or loss of nutrition, want of development, and those which are due to disease altering the physical and chemical characters of the tissue. When the animal can be examined both before and after slaughter, and the symptoms of the living animal can be compared with the lesions of the cadaver, the recognition of the pernicious changes is usually easy ; but when the animal has been slaughtered at a distance and only the diseased meat can be inspected, it frequently becomes difficult to determine the degree of alteration. Contagious pleuro-pneumonia should only be allowed to condemn meat when the animal has an acute fever and the meat is bloody; when the animal has a chronic inflammation, especially diarrhœa, and when the meat is emaciated; when the animal has gangrenous spots and the meat is septic. The presence of tuberculosis in an animal should condemn all its flesh, but practically only does when the disease is generalized, affects the abdominal viscera and the lymphatic glands of the whole body. When only the lungs are found affected and the lymphatic's lying under the heads of the ribs do not show lesions, the meat is usually marketable.

With the Hebrew butcher, however, any evidence of tuberculosis condemns it as Jewish food, and transfers it to the neighboring more lenient stall of the Christian butcher. Any cause which decomposes the fat on the ends of the cut bones or on the surface of the meat favors the beginning of decomposition and attracts flies, and septic germ's to hasten putrefaction. The essayist went very minutely into the anatomy of the bullock to choose choice meats from and the reasons therefore. The paper was able and should be in print, it received much applause. Dr. P. S. Keyoes of the State Board of Health, said that there was no meat inspection in the city, but that the milk inspection was progressing very favorably, under the very weak law that we have, and of which dealers will take advantage. Dr. A.W. Clement, of Baltimore, spoke of the progress in meat inspection in Montreal,

Canada, where for a time, strictness was required of the butchers, but insufficient appropriation allowed of nothing of a permanent character.

He showed the need of public abattoirs, the want of which was a cause for the evil system prevailing. In Baltimore especially, he said, there was a lack of any proper inspection of milk or meat. Dr. W. E. B. Miller, of the Bureau of Animal Industry, referred to the restrictions enforced by Great Brittian on animals shipped from their ports, and thought that a careful examination would contribute to the raising of the embargo. Since Nov. 1st. 10,781 cattle had been examined here, of which number 144 were diseased and 123 had tuberculosis. He has discovered that the meat of tuberculous animals is largely used in the lower section of the city for making bologna sausages. Dr. Miller stated that in New Jersey the number of tuberculous animals is on the increase. Dr. W. B. Werntz, stated that when he was a Member of Councils he succeeded in having a system of inspection passed. The inspector was called a Market Clerk, and inspected the meats and the weights and measures. But through the efforts of farmers' and market people they had been abolished by an act of the legislature, since which time, we have no standard of weights in our market, nor any inspection of meat. This action on the part of the legislature was approved by our present Governor. Dr. J. Cheston Morris referred to the work of the milk inspector here, whom he considered as well qualified, but who, in order to attain the best results, must be enabled to visit the dairies, and there examine the animals from which milk is sold. Dr. F. Bridge believed that every herd and every dairy in the State ought to be inspected. The inspection in this State is deficient for the reason that while there are about $90,000,000 worth of cattle, the entire appropriation for their inspection amounts to $2,500.

Mr. George Abbott, a prominent milk dealer, being called upon for his views, expressed pleasure that this subject was brought up for discussion and action. Physicians, said he, want 50 per cent. or more water added to the milk for the use of infants. This he said is not a matter of much concern, but the removal of cream is, as the latter could not be readily detected. The speaker thought that statements made in the *Press* were calculated to prejudice the people against the present milk supply. He knew of men who would not keep a tuberculous cow were the fact shown to them. He would welcome the passage, by the legislature, of a bill enforcing inspection of herds and guarding the public against all risks.

Dr. J. W. Gadsden, introduced as a veteran in this branch of veterinary science, expressed his opinion about tuberculosis which he said was found in animals largely in Illinois, Pennsylvania and other States. He individually did not drink milk at his home except when boiled. He said England would not raise the embargo on American cattle while a single case of pleuro-pneumonia exists in the United States.

After further discussion the subject was on motion of Dr. Huidekoper continued for another meeting.

On motion adjourned.

W. S. Kooker, *Secretary*, Pro Tem.

*Keystone Veterinary Medical Association.*—The regular meeting of the Keystone Veterinary Medical Association was called to order at the College of Physicians, Philadelphia, on April 4, at 8 P. M. Dr. Hoskins in the chair. The following members answered roll call :

Drs. Bridge, Weber, Hoskins, Goentner and Drake. The minutes of previous meeting were read and adopted. The President in absence of a quorum of the Board of Trustees appointed Drs. Goentner and Drake, to act with Dr. Weber on the resignation of Drs. W. L. Zuill, W. H. Ridge, Chas. S. Williams and C. H. Magill.

The Board reported as follows : We hereby report that we recommend the acceptance of the resignations of Drs. W. L. Zuill, W. H. Ridge, Chas. S. Williams and C. H. Magill. Signed, S. E. Weber, M. W. Drake and Chas. T. Goentner.

It was moved and seconded the above be adopted. Carried.

Dr. Hoskins asked if mares have an increase in temperature during the period of gestation. Dr. Bridge answered that he had noticed an increase of two degrees for the first two months, Dr. Hoskins stated that at seven months his mare had a temperature of 101 1-5 and continued about the same until parturition. Dr. Bridge said cattle always have a rise of temperature during gestation.

Dr. W. H. Hoskins read a paper entitled "The Necessity of a Uniform Standard aud Title of Veterinary Medicine in America," (Vide page 209.)

The essayist desired that the Keystone Association should be among the first to advise a single standard of examinations in veterinary medicine, and placing itself on record in this direction; he thought other associations would do the same and the colleges would ultimately make recognition of it and extend their course to at least three sessions. Dr. Goentner was doubtful if we could in any way influence the schools. Dr. Glass, who had been called to the chair, took this opportunity of assuring the association, that the Veterinary Department of the University of Pennsylvania, was always desirious of retaining the good will of the profession and did not wish at any time or in any way to run counter to the best views of veterinary associations.

The recent move taken by the officials of that Institution was purely a personal matter and not a college feeling.

Dr. Weber was glad that the Keystone Veterinary Medical Association was the first to advise the adoption of these measures, but could not see how it can be carried successfully to an end. And in recognizing the higher standard of the University of Pennsylvania, wished that others should have the same. He asked, are the graduates of Agricultural Colleges recognized by the United States Veterinary Medical Association as regular graduates? Dr. Hoskins answered him yes, that they were admitted to membership at Chicago, September 17, 1891.

Dr. Bridge thinks the step a desirable one but difficult for the association to express its opinion to the colleges for a higher standard of requirements.

Dr. Weber advised the State joining the Colleges in taking this step.

Dr. Goentner recommends a body of qualified men to start a National Board of Examiners and not to allow recent graduates to enter unless they can pass the Board, if the different associations would join and work together the point might be attained.

Dr. Hoskins said the people can be reashed through public opinion; that the schools will not listen to the Keystone Veterinary Medical Association but would if all the societies would join and work for the same point. If the Keystone Veterinary Medical Association would adopt a rule and not admit a member unless a three years' course had been pursued he would vote for it.

Dr. Glass gave his views of the matter, that it should be a National Board of Examiners and not a State matter, as it would get into politics and favors be shown in granting the positions. We should get the veterinarians at large interested, to push it and keep at it.

Dr. Glass then explained the numbers of the University of Pennsylvania graduates as smaller compared with the Canadian schools to be the result of the three years' course. He thought it would be adviseable to stamp out all the schools and form a limited number of Government Schools the same as in Germany inasmuch as the veterinarian is the preventor, and the physician, the cures of disease.

Dr. Goentner made a motion that a committee be appointed to draft suitable resolutions, embodying the views of the Keystone Veterinary Medical Association on the subject of a higher standard and a single board of examiners. Seconded by Dr. Hoskins. Passed.

The President appointed the following committee: Drs. Glass, Chairman; Goentner and Weber. Election of officers; the secretary's place now being vacant, Drs. Kooker and Drake were proposed; Dr. Kooker's name being withdrawn at his own request, Dr. Drake was elected by acclamation.

The meeting then adjourned.

W. H. Hoskins, *President.* M. W. Drake, *Secretary.*

